# African Ancestry and Its Correlation to Type 2 Diabetes in African Americans: A Genetic Admixture Analysis in Three U.S. Population Cohorts

**DOI:** 10.1371/journal.pone.0032840

**Published:** 2012-03-16

**Authors:** Ching-Yu Cheng, David Reich, Christopher A. Haiman, Arti Tandon, Nick Patterson, Selvin Elizabeth, Ermeg L. Akylbekova, Frederick L. Brancati, Josef Coresh, Eric Boerwinkle, David Altshuler, Herman A. Taylor, Brian E. Henderson, James G. Wilson, W. H. Linda Kao

**Affiliations:** 1 Department of Epidemiology, Johns Hopkins University, Baltimore, Maryland, United States of America; 2 Welch Center for Prevention, Epidemiology and Clinical Research, Johns Hopkins University, Baltimore, Maryland, United States of America; 3 Department of Medicine, Johns Hopkins University, Baltimore, Maryland, United States of America; 4 Saw Swee Hock School of Public Health, and Department of Ophthalmology, Yong Loo Lin School of Medicine, National University of Singapore, Singapore, Singapore; 5 Duke-NUS Graduate Medical School, Singapore, Singapore; 6 Singapore Eye Research Institute, Singapore, Singapore; 7 Department of Genetics, Harvard Medical School, Boston, Massachusetts, United States of America; 8 Department of Medicine, Harvard Medical School, Boston, Massachusetts, United States of America; 9 Department of Preventive Medicine, Keck School of Medicine, University of Southern California, Los Angeles, California, United States of America; 10 Program in Medical and Population Genetics, Broad Institute of Harvard and M.I.T., Cambridge, Massachusetts, United States of America; 11 Jackson Heart Study Analysis Group, Jackson State University, Jackson, Mississippi, United States of America; 12 Human Genetics Center, University of Texas Health Science Center at Houston, Houston, Texas, United States of America; 13 Center for Human Genetic Research and Diabetes Unit, Department of Medicine, Massachusetts General Hospital, Boston, Massachusetts, United States of America; 14 Jackson State University, Tougaloo College, and the University of Mississippi Medical Center, Jackson, Mississippi, United States of America; 15 Department of Physiology and Biophysics, The University of Mississippi Medical Center, Jackson, Mississippi, United States of America; Postgraduate Medical Institute & Hull York Medical School, University of Hull, United Kingdom

## Abstract

The risk of type 2 diabetes is approximately 2-fold higher in African Americans than in European Americans even after adjusting for known environmental risk factors, including socioeconomic status (SES), suggesting that genetic factors may explain some of this population difference in disease risk. However, relatively few genetic studies have examined this hypothesis in a large sample of African Americans with and without diabetes. Therefore, we performed an admixture analysis using 2,189 ancestry-informative markers in 7,021 African Americans (2,373 with type 2 diabetes and 4,648 without) from the Atherosclerosis Risk in Communities Study, the Jackson Heart Study, and the Multiethnic Cohort to 1) determine the association of type 2 diabetes and its related quantitative traits with African ancestry controlling for measures of SES and 2) identify genetic loci for type 2 diabetes through a genome-wide admixture mapping scan. The median percentage of African ancestry of diabetic participants was slightly greater than that of non-diabetic participants (study-adjusted difference = 1.6%, *P*<0.001). The odds ratio for diabetes comparing participants in the highest vs. lowest tertile of African ancestry was 1.33 (95% confidence interval 1.13–1.55), after adjustment for age, sex, study, body mass index (BMI), and SES. Admixture scans identified two potential loci for diabetes at 12p13.31 (LOD = 4.0) and 13q14.3 (Z score = 4.5, *P* = 6.6×10^−6^). In conclusion, genetic ancestry has a significant association with type 2 diabetes above and beyond its association with non-genetic risk factors for type 2 diabetes in African Americans, but no single gene with a major effect is sufficient to explain a large portion of the observed population difference in risk of diabetes. There undoubtedly is a complex interplay among specific genetic loci and non-genetic factors, which may both be associated with overall admixture, leading to the observed ethnic differences in diabetes risk.

## Introduction

Approximately 13% of the U.S. adults have type 2 diabetes [1], representing a significant burden on public health in the United States. Type 2 diabetes is approximately twice as prevalent in African Americans as in European Americans. In the Multiethnic Cohort (MEC), this racial/ethnic difference persisted after stratification by body mass index (BMI) [2]. Data from the National Health and Nutrition Examination Survey (NHANES) confirm the substantial racial disparity in diabetes across the U.S. [1], [3]. In the Atherosclerosis Risk in Communities (ARIC) Study, African Americans are twice as likely as whites to develop incident type 2 diabetes—a disparity which persists even after extensive adjustment for socioeconomic status (SES) and behavioral risk factors [4]. This persistent disparity suggests that genetic factors may contribute to ethnic differences in susceptibility to type 2 diabetes.

Despite remarkable efforts in the past three years that have led to the discovery of more than 30 susceptibility loci for type 2 diabetes and related quantitative traits [5]–[18], there has been only one genome-wide association study of type 2 diabetes in African Americans [19]. The disparity in diabetes prevalence between Americans of African and European ancestry makes diabetes an attractive phenotype to study by admixture mapping, a method that systematically scans the genomes of groups of recently admixed individuals (e.g., African Americans) to search for genetic loci where persons with a disease or trait, in aggregate, have more (or less) African ancestry than their genome-wide average. Admixture mapping and subsequent fine-mapping studies have been successful in identifying genetic variants for other complex phenotypes, including prostate cancer [20], [21], end stage renal disease [22], white blood cell count [23], [24], and circulating levels of interleukin 6 soluble receptor [25].

Given the observed ethnic/racial disparities in diabetes prevalence, we hypothesized that some diabetes susceptibility alleles are present at higher frequency in African Americans than in European Americans, resulting in association between genetic ancestry and diabetes risk that is independent of its association with other non-genetic risk factors for type 2 diabetes. Thus we sought 1) to establish the association of genetic ancestry with diabetes and related quantitative traits in African Americans, after accounting for the non-genetic risk factors, and 2) to identify diabetes susceptibility loci by conducting a genome-wide admixture mapping scan. To maximize power to detect genetic association, we performed a pooled analysis of 7,021 African-American participants, including 2,373 diabetic cases, from three U.S. population cohorts, including the ARIC Study, the Jackson Heart Study (JHS), and the MEC study.

## Results

### Characteristics of participants

The characteristics and genetic ancestry of the 7,021 African Americans (including 2,373 with type 2 diabetes) included in the study are shown in Table 1 and Figure S1. The overall median global African ancestry was 83.7% (interquartile range, 76.2%–88.7%). African ancestry distributions were different among the three cohorts, with MEC participants having a lower average percentage of African ancestry (*P*<0.001). Diabetic participants tended to have higher BMI, lower education level and lower family income, compared to non-diabetic participants (Table S1).

**Table 1:** Genetic African ancestry by participant characteristics and study.

	ARIC			JHS			MEC		
Characteristic	No. (%)	African Ancestry, Median (IQR), %	*P* Value^a^	No. (%)	African Ancestry, Median (IQR), %	*P* Value^a^	No. (%)	African Ancestry, Median (IQR), %	*P* Value^a^
Overall	2285 (100)	84.9 (77.8–89.5)		3185 (100)	84.0 (77.9–88.7)		1551 (100)	80.6 (69.8–87.4)	
Age, y									
21–39	0 (0)			237 (7.4)	84.1 (79.4–87.9)		0 (0)		
40–59	1741 (76.2)	85.0 (78.2–89.4)	0.872	1519 (47.7)	83.9 (77.9–88.3)	0.455	627 (40.4)	82.0 (72.4–87.9)	<0.001
≥60	544 (23.8)	84.6 (76.4–89.9)		1429 (44.9)	84.2 (77.4–89.2)		924 (59.6)	79.3 (67.5–86.9)	
Gender									
Men	918 (40.2)	85.1 (78.5–89.6)	0.187	1210 (38.0)	83.8 (77.6–88.2)	0.100	1001 (64.5)	79.2 (67.5–86.7)	<0.001
Women	1367 (59.8)	84.7 (77.4–89.3)		1975 (62.0)	84.2 (78.2–88.8)		550 (35.5)	82.2 (73.7–88.3)	
BMI, kg/m^2^									
<25	530 (23.2)	83.9 (76.8–89.0)		403 (12.6)	84.0 (78.2–88.8)		277 (17.8)	78.9 (67.9–85.8)	
25–<30	865 (37.9)	84.1 (76.8–89.2)	<0.001	1053 (33.1)	83.8 (77.0–88.5)	0.286	682 (44.0)	79.2 (67.2–87.2)	<0.001
≥30	890 (38.9)	85.9 (79.1–89.9)		1729 (54.3)	84.3 (78.3–88.7)		592 (38.2)	82.1 (73.4–88.3)	
Diabetes									
Yes	631 (27.6)	85.7 (79.3–89.5)	0.008	829 (26.0)	85.3 (79.5–89.3)	<0.001	913 (58.9)	81.3 (71.8–88.1)	<0.001
No	1654 (72.4)	84.6 (77.0–89.4)		2356 (74.0)	83.6 (77.3–88.3)		638 (41.1)	79.4 (66.2–86.1)	

ARIC, the Atherosclerosis Risk in Communities Study; JHS, the Jackson Heart Study; MEC, the Multiethnic Cohort; IQR, interquartile range; BMI, body mass index (calculated as weight in kilograms divided by height in meters squared).

a *P* value was generated from the Wilcoxon rank-sum test or the Kruskal-Wallis test.

### Association of ancestry with diabetes

Pooling the three cohorts together, the median percentage of African ancestry of diabetic participants was 1.6% greater than that of non-diabetic participants (*P*<0.001, adjusted for study). The odd ratios (ORs) for diabetes were higher with increasing tertiles of African ancestry (*P* for trend<0.001, Model 1 in Table 2) after adjustment for age, sex, and study. With additional adjustment for BMI, individuals in the second and third tertiles were still, respectively, 1.21 and 1.4 times more likely to have diabetes than their counterparts in the first tertile (Model 2 in Table 2).

**Table 2:** Odds ratio of diabetes by genetic African ancestry.

	African Ancestry				Excess Odds Explained, %^a^	
Study and Model	Tertile 1	Tertile 2	Tertile 3	*P* Value for Trend	Tertile 2	Tertile 3
ARIC, JHS and MEC combined						
African ancestry, %	<79.4	79.4–87.2	>87.2			
Diabetes, yes/no, No.	774/1574	763/1612	836/1462			
Model 1, base^b^	1 [Reference]	1.25 (1.10–1.43)^f^	1.48 (1.29–1.69)^g^	<0.001	[Reference]	[Reference]
Model 2, BMI^c^	1 [Reference]	1.21 (1.06–1.39)^f^	1.40 (1.22–1.61)^g^	<0.001	16.0	16.7
ARIC and JHS combined						
African ancestry, %	<80.5	80.5–87.5	>87.5			
Diabetes, yes/no, No.	296/1088	255/1000	406/962			
Model 1, base^b^	1 [Reference]	1.35 (1.16–1.57)^g^	1.47 (1.26–1.71)^g^	<0.001	[Reference]	[Reference]
Model 2, BMI^c^	1 [Reference]	1.32 (1.17–1.70)^f^	1.40 (1.20–1.64)^g^	<0.001	8.6	14.9
Model 3, SES^d^	1 [Reference]	1.27 (1.09–1.49)^f^	1.37 (1.17–1.59)^g^	<0.001	22.9	21.3
Model 4, BMI+SES^e^	1 [Reference]	1.26 (1.07–1.48)^f^	1.33 (1.13–1.55)^g^	<0.001	25.7	29.8

ARIC, the Atherosclerosis Risk in Communities Study; JHS, the Jackson Heart Study; MEC, the Multiethnic Cohort; BMI, body mass index (calculated as weight in kilograms divided by height in meters squared); SES, socioeconomic status (including education, income and occupation).

a Excess risk explained is defined as (*θ*_1_−*θ*_2_)/(*θ*_1_−1) where *θ*_1_ is the odds ratio of diabetes due to increase in African ancestry in Model 1; *θ*_2_ is the odds ratio after additional adjustment for covariates in each model; and *θ*_1_−1 is the excess odds of diabetes due to increase in African ancestry.

b Model 1: odds ratio (95% confidence interval) is adjusted for age, sex, and study.

c Model 2: odds ratio (95% confidence interval) is adjusted for covariates in Model 1 and BMI.

d Model 3: odds ratio (95% confidence interval) is adjusted for covariates in Model 1 and SES.

e Model 4: odds ratio (95% confidence interval) is adjusted for covariates in Model 1, BMI, and SES.

f *P*<0.05, as compared to the reference tertile.

g *P*<0.001, as compared to the reference tertile.

**Figure 1:**
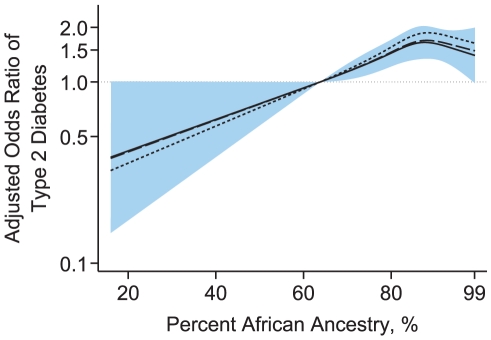
Odds ratio of type 2 diabetes by African ancestry in the ARIC and JHS studies. Odds ratios were based on restricted cubic splines with knots at the 5th, 35th, 65th and 95th percentiles. The reference was set at the 5th percentile (63.8%) of the African ancestry distribution. The odds ratio was adjusted for age, sex and study (shot-dashed line), and further adjusted for socioeconomic status, including education level, family income, and occupations (long-dashed line). The solid line indicates the odds ratio adjusted for age, sex, study, socioeconomic status, and BMI; the shaded area represents its 95% confidence intervals.

To determine whether the observed excess odds of diabetes with increasing African ancestry might further be explained through the association between genetic ancestry and other non-genetic risk factors, such as measures of SES, we constructed additional models using only ARIC and JHS, where these data were available. Univariately, measures of SES were associated with both genetic ancestry (Table S2) and diabetes (Table S3). Even after adjusting for the three SES indicators, individuals in the second and third tertiles of African ancestry were about 1.27 and 1.37 times more likely to have diabetes than those in the first tertile (Model 3 in Table 2). The three SES indicators together accounted for about 22% of the excess odds of diabetes with increasing African ancestry. Collectively, BMI and the three SES measures explained about 30% of the excess odds of diabetes observed in individuals in the third tertile. The associations between increasing African ancestry and greater odds of diabetes were also evident in models based on restricted cubic splines (Figure 1).

### Association of ancestry with diabetes-related traits

In ARIC and JHS only, we assessed the association of African ancestry to hemoglobin A_1c_ (HbA_1c_), fasting glucose and insulin level, and insulin resistance, which was estimated by the homeostasis model assessment (HOMA-IR) (Table S4 and Table S5). A total of 4,880 participants had measurements on HbA_1c_, and 5,037 had fasting glucose and insulin level. Greater African ancestry was significantly correlated with higher HbA_1c_ (*P*<0.001, see also Figure S2). However, African ancestry accounts for only 0.7% of the variance in HbA_1c_ levels after adjustment for age, sex and study, while SES alone accounted for a slightly higher proportion of variance of 1.1%. After excluding individuals who were receiving diabetes treatment (because such treatment directly affects trait levels), the effects of ancestry generally became weaker (Table 3). The other three traits, fasting glucose, insulin and HOMA-IR levels, were also positively associated with African ancestry before and after adjustment, but the associations were not statistically significant (Table 3 and Table S5).

**Table 3:** Mean difference in the levels of diabetes-related quantitative traits by genetic African ancestry after excluding participants receiving diabetes treatment.

Trait and Model	African Ancestry^a^			*P* Value for Trend	Effect Explained, %^b^	
	Tertile 1	Tertile 2	Tertile 3		Tertile 2	Tertile 3
Hemoglobin A_1c_, % (n = 4100)						
Model 1, base^c^	0 [Reference]	0.10 (0.02–0.18)^g^	0.10 (0.02–0.18)^g^	0.011	[Reference]	[Reference]
Model 2, BMI^d^	0 [Reference]	0.08 (0.01–0.16)^g^	0.09 (0.01–0.17)^g^	0.028	20.0	10.0
Model 3, SES^e^	0 [Reference]	0.07 (−0.01–0.16)	0.07 (−0.01–0.16)	0.073	30.0	30.0
Model 4, BMI+SES^f^	0 [Reference]	0.06 (−0.01–0.14)	0.06 (−0.02–0.14)	0.122	40.0	40.0
Glucose, mg/dL (n = 4423)						
Model 1, base^c^	0 [Reference]	1.20 (−0.89–3.28)	1.57 (−0.51–3.65)	0.139	[Reference]	[Reference]
Model 2, BMI^d^	0 [Reference]	0.93 (−1.14–2.99)	1.16 (−0.91–3.22)	0.272	22.5	26.1
Model 3, SES^e^	0 [Reference]	0.48 (−1.62–2.58)	0.73 (−1.38–2.84)	0.501	60.0	53.5
Model 4, BMI+SES^f^	0 [Reference]	0.30 (−1.80–2.38)	0.41 (−1.68–2.51)	0.700	75.8	73.9
Insulin, mU/L (n = 4423)						
Model 1, base^c^	0 [Reference]	0.87 (0.12–1.62)^g^	0.68 (−0.07–1.43)	0.073	[Reference]	[Reference]
Model 2, BMI^d^	0 [Reference]	0.58 (−0.11–1.27)	0.23 (−0.46–0.93)	0.503	33.3	66.2
Model 3, SES^e^	0 [Reference]	0.81 (0.06–1.57)^g^	0.60 (−0.16–1.36)	0.125	6.9	11.8
Model 4, BMI+SES^f^	0 [Reference]	0.60 (−0.10–1.29)	0.25 (−0.46–0.95)	0.498	31.0	63.2
HOMA-IR (n = 4423)						
Model 1, base^c^	0 [Reference]	0.28 (0.05–0.52)^g^	0.23 (−0.00–0.47)	0.052	[Reference]	[Reference]
Model 2, BMI^d^	0 [Reference]	0.20 (−0.02–0.42)	0.10 (−0.12–0.32)	0.352	28.6	56.5
Model 3, SES^e^	0 [Reference]	0.24 (−0.00–0.47)	0.17 (−0.07–0.41)	0.159	14.3	26.1
Model 4, BMI+SES^f^	0 [Reference]	0.17 (−0.05–0.40)	0.07 (−0.15–0.30)	0.536	39.3	69.6

BMI, body mass index (calculated as weight in kilograms divided by height in meters squared); SES, socioeconomic status (including education, income and occupation).

a Tertiles 1, 2 and 3 of African ancestry are <80.3%, 80.3%–87.3% and >87.3%, respectively, for hemoglobin A_1c_, and <80.2%, 80.2%–87.3% and >87.3%, respectively, for glucose, insulin, and HOMA-IR.

b Effects explained is defined as (β_1_−β_2_)/β_1_ where β_1_ is the regression coefficient of traits in Model 1; β_2_ is the regression coefficient after adjustment for covariates in each model.

c Model 1: Mean difference (95% confidence interval) is adjusted for age, sex, and study.

d Model 2: Mean difference (95% confidence interval) is adjusted for covariates in Model 1 and BMI.

e Model 3: Mean difference (95% confidence interval) is adjusted for covariates in Model 1 and SES.

f Model 4: Mean difference (95% confidence interval) is adjusted for covariates in Model 1, BMI, and SES.

g *P*<0.05, as compared to the reference tertile.

### Admixture scans

**Figure 2:**
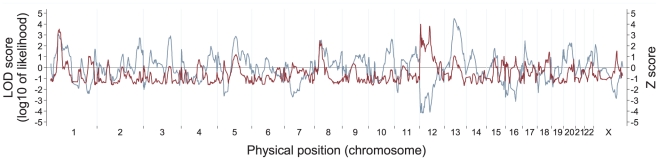
Admixture scans for genetic loci of type 2 diabetes in African Americans. Locus-genome statistic (LOD score, red line) and case-control statistic (Z score, blue gray line) are shown. A signal was detected at 12p13.31 with a locus-specific LOD score of 4.0, just reaching the threshold of 4 for suggestiveness. The 12p13.31 peak was also supported by the case-control statistic (Z score = −4.2, nominal *P* = 3.3×10^−5^). The second strongest admixture signal was observed on the same chromosome at 12q13.13 (locus-specific LOD = 3.8). There was also an admixture peak at 13q14.3 that did not reach genome-wide significance by the locus-genome statistic (locus-specific LOD = 1.1), but that had the largest magnitude case-control Z score anywhere in the genome (Z score = 4.5, nominal *P* = 6.6×10^−6^).

We conducted genome-wide admixture scans on type 2 diabetes in the 7,021 African Americans (Figure 2 and Table S6). In the diabetic cases, we detected an admixture association in diabetic cases at 12p13.31 with a locus-specific LOD of 4.0, just reaching the threshold for suggestiveness. The 12p13.31 peak was also supported by a case-control Z score of −4.2 (nominal *P* = 3.3×10^−5^), which was marginally genome-wide significant. At this locus, diabetic cases had lower European ancestry (i.e., higher African ancestry) than non-diabetic controls. The second strongest admixture signal was observed nearby at 12q13.13 (locus-specific LOD = 3.8), and the third was at 1p33 (locus-specific LOD = 3.5). There were no other loci with LOD scores >2.5. Averaging the LOD scores across all loci in the genome, we obtained a genome-wide score of 1.5, again reaching the threshold of 1 for suggestiveness. Interestingly, at 13q14.3 the LOD scores was far from significant (locus-specific LOD = 1.1), but this locus had both the largest magnitude (either positive or negative) case-control statistic anywhere in the genome (Z score = 4.5, nominal *P* = 6.6×10^−6^), exceeding the level of nominal genome-wide significance. At the 13q14.3 locus, the diabetic cases had higher European ancestry than the non-diabetic controls.

## Discussion

We have conducted a large-scale admixture genetic analysis in more than 7,000 African Americans to determine the association of African ancestry with type 2 diabetes and to map susceptibility loci for type 2 diabetes. With 2,373 cases with type 2 diabetes and 4,648 controls, we found that greater African ancestry was significantly associated with type 2 diabetes and HbA_1c_ values even after adjustment for BMI and markers of SES, including education, income, and occupation. Despite the significant association between greater African ancestry and type 2 diabetes, no major locus for diabetes could be detected by our admixture scans, using the more powerful locus-genome statistic.

Our results show that there is 30% to 40% increase in odds of type 2 diabetes among participants in the highest (>87.5%) vs. the lowest (<80.5%) tertile of African ancestry, even after adjustment for measures of SES and/or BMI. Our restricted cubic spline models also support this extrapolation, implying that genetic ancestry is a major independent determinant of the observed disparity in diabetes risk between the two ethnic groups. We note that in our study, markers of SES (education, income, and occupation) account for only a modest proportion (∼22%) of the excess odds of type 2 diabetes due to ancestry. The results contrast to previous findings in Hispanic Americans [26], where the association between their non-European ancestry and type 2 diabetes is also significant, but where SES appears to be a much greater confounder, as adjustment for it significantly attenuated the association signal. It is also worth noting that the previous study had less power than our study as it used fewer individuals and fewer ancestry informative markers [26].

Insulin resistance and β–cell dysfunction are known to be major factors in the pathogenesis of type 2 diabetes. Evidence from epidemiological studies indicates that African Americans tend to be more insulin resistant and have greater insulin responses to glucose than European Americans [27]–[33]. Our results showed a positive (but statistically non-significant) correlation between African ancestry and HOMA-IR, in line with one earlier study that demonstrated that children with greater African ancestry had lower insulin sensitivity and a higher acute insulin response [34]. In genome-wide scans to date, the majority of the genetic variants for type 2 diabetes identified in European-derived populations appeared to be related to impaired insulin secretion [6], [17], [35], while only *IRS1* has been unequivocally associated with insulin resistance [18]. However, a limitation is that these studies have been carried out largely in Europeans. It will be interesting to explore whether the loci associated to type 2 diabetes in African Americans are also associated with impaired insulin secretion, once genome-wide association studies of sufficient power are carried out.

Previous genome-wide scans for type 2 diabetes in African-descent populations have been extremely limited, and there has only been one study using admixture-based approaches. In the GENNID (Genetic of NIDDM) Study, using markers from a linkage panel in 1,450 African Americans, the strongest admixture association was found on chromosome 12 (90 cM), but no loci achieved genome-wide significance [36]. In our large population with a high number of ancestry informative markers, the two most interestingly loci using were at 12p13.31 and 13q14.3. While neither of these loci was significant by our locus-genome statistic which has the most statistical power, the case-control Z score at both loci exceeded the threshold for genome-wide significance, which makes these loci of interest for further study. An attractive candidate gene at the 12p13.31 locus is glyceraldehyde-3-phosphate dehydrogenase (*GAPDH*), which is a key enzyme in the glycolytic pathway and is known to affect insulin receptor signaling [37]. The 12p13.31 locus has been found to be associated with type 1 diabetes in previous genome-wide association studies in European-derived populations [15], but neither of the two loci has been associated with type 2 diabetes in either African Americans or Europeans.

An interesting feature of our admixture scanning results is that diabetes risk at 12p13.31 and 13q14.3 were associated with ancestry in opposite directions. At 13q14.3, greater European ancestry is associated with a higher risk of diabetes, opposite to the direction of the overall epidemiological association, a phenomenon that we documented for the first time in a study of obesity loci [38]. These two loci, together with the other modest admixture signals on chromosome 12 and 1, and the absence of significant signals in locus-genome statistic elsewhere in the genome, suggest no evidence for a large genetic effect for type 2 diabetes that is racially/ethnically differentiated, such as that at the *MYH9*/*ApoL1* locus for non-diabetic end stage renal disease [22], [39]. Thus multiple loci modest effects may, in aggregate, explain the apparent difference in genetic risk for type 2 diabetes between African Americans and European Americans.

Our study has important limitations. Despite the fact that our study size far exceeds previous genome scans for type 2 diabetes risk loci in African Americans, statistical power remains an important concern. We carried out simulation studies to examine the power of our study to detect a genomic locus of elevated African ancestry [40]. With a total of 2,373 diabetic cases, we expect to have 80% power to detect a 1.8-fold increased risk of type 2 diabetes per allele for alleles that are ancestry informative between Europeans and West Africans but less power for weaker ORs. A second limitation is that we used BMI as the only measure of adiposity. Including some other measure, such as waist circumference, might further attenuate the diabetes-ancestry association. In our previous analysis, however, we found that BMI, but not waist circumference, was significantly correlated with genetic ancestry after adjustment for SES [41], suggesting that confounding by waist circumference would have a minimal effect on results. Third, as in many studies involving SES, we were not able to fully assess SES and made inferences about SES based on education, income, and occupation, which, although are strong markers for SES, are still imperfect [42]. For example, SES may also be correlated with other diabetes risk factors, such as diet and life-style, and historical socioeconomic factors could in theory interact in complex ways with African ancestry to influence diabetes risk, making the associations among them even more complicated.

In summary, in community-based populations with more than 7,000 African Americans, we found that genetic ancestry is significant associated with type 2 diabetes above and beyond the effects of markers of SES, and we detected several suggestive loci that may harbor genetic variants modulating diabetes risk. These results suggest that in African Americans, genetic ancestry has a significant effect on the risk of type 2 diabetes that are independent of the contribution of SES, but that no single locus with a major effect explains a large portion of the observed disparity in diabetes risk between African Americans and European Americans. In addition, they suggest that genetic measured African ancestry contributes to the risk of type 2 diabetes via both genetic and non-genetic pathways. The effect of ancestry on any individual locus in the genome is likely to be modest, but in aggregate, differences in ancestry may contribute substantially to the observed ethnic disparity in risk of type 2 diabetes.

## Materials and Methods

### Ethics statement

This study was conducted according to the principles expressed in the Declaration of Helsinki. All data collections were carried out according to protocols approved by Johns Hopkins Bloomberg School of Public Health Institutional Review Board for the study of human subjects. Written informed consent was obtained from all participants.

### Study populations

The individuals enrolled in the present study came from three studies: the ARIC, JHS and MEC studies (Table 1). A detailed description of the three studies as well as the numbers of participants that were analyzed after applying various data quality filters are presented in Text S1. A brief description of each study is provided here.

The ARIC study is a prospective epidemiologic study that examines clinical and subclinical atherosclerotic disease in a cohort of 15,792 persons, including 4,266 self-reported African Americans, aged 45 to 64 years at their baseline examination from 1987 to 1989. The sampling procedure and methods used in ARIC have been described in detail elsewhere [43]. A total of 2,285 African-American participants from the ARIC study were included in the current analysis.

The JHS is a long-term, community-based observational study of cardiovascular disease and its risk factors in 5,301 self-identified African Americans recruited between 2000 and 2004 from three counties surrounding Jackson, Mississippi [44], [45]. Unrelated persons aged 35–84 were enrolled, and the remaining participants, at least 21 years old, were members of the nested JHS Family Study [46]. A total of 3,185 participants from the JHS were included in the current study.

The MEC study is a prospective cohort of 215,251 individuals recruited between 1993 and 1996 in Hawaii and Los Angeles, California, of whom 16.3% were African Americans [47]. Potential cohort members were identified primarily through Department of Motor Vehicles drivers' license files and, additionally for African Americans, Health Care Financing Administration data files. Participants were between the ages of 45 and 75 years at the time of recruitment. A total of 1,551 African-American participants from the MEC study, selected for a diabetes case-control study, were included in this analysis.

### Diabetes and related traits

Information on body weight and height was collected in all three studies. In ARIC and JHS, anthropometry was performed during the clinical visit in the fasting state with an empty bladder by certified technicians. Body mass index was calculated as weight (in kg)/height (in meters) squared. In MEC, BMI was calculated using self-reported weight and height. The ARIC Study and JHS also have measurements of other diabetes-related quantitative traits, including fasting serum glucose and insulin concentrations, and HbA_1c_. Participants were asked to fast for at least 12 hours before morning blood collection. Blood samples were collected into vacuum tubes containing serum-separator gel (glucose, insulin) or EDTA (HbA_1c_). Specimens were then processed and analyzed in the ARIC and JHS Central Laboratories at University of Minnesota [48], [49]. Serum glucose and insulin were measured by the hexokinase method [49], [50] and by radioimmunoassay [49], respectively. HbA_1c_ was assayed with Tosoh HPLC instruments [48], [49]. The present analysis used data from the baseline examination in all three cohorts, except that in ARIC HbA_1c_ was measured in stored whole blood samples from the second clinical visit [48]. Insulin resistance was estimated by the homeostasis model assessment (HOMA-IR) as fasting plasma glucose [mmol/l] times fasting serum insulin [mU/L] divided by 22.5.

Type 2 diabetes was defined as the presence of any one of the following at the baseline examination in the ARIC and JHS studies: 1) fasting glucose ≥7.0 mmol/l (126 mg/dl); 2) non-fasting glucose ≥11.1 mmol/l (200 mg/dl); 3) hemoglobin A_1c_ ≥6.5% [51]; 4) current use of diabetic medication; or 5) a positive response to the question “Has a doctor ever told you that you had diabetes (sugar in the blood)?” In addition, diabetic individuals in ARIC or JHS who reported age of diagnosis younger than 30 years were excluded. In the MEC, diabetic individuals were defined as those who indicated on the baseline or follow-up questionnaires that they had a history of diabetes, and were taking medication for diabetes at the time of blood draw. The question did not differentiate between type 1 diabetes and type 2 diabetes, and thus we expect a small fraction (<10%) of the respondents to have type 1 diabetes [52]. In the ARIC and JHS study, non-diabetic controls were defined as African-American participants who did not have diabetes and were derived from the same populations as the diabetic cases. In the MEC, non-diabetic controls were from a group of samples who neither had a history of diabetes nor were taking medication for diabetes and had been specifically genotyped as part of previous admixture scans for prostate cancer [20] and hypertension [53].

### Socioeconomic status

Information on three SES indicators, including personal education level, occupation, and family income, was collected during the baseline interview in the ARIC and JHS study. For the purpose of this analysis, education level was categorized into four groups: 1) less than high school; 2) high school graduate or high school-level General Educational Development credential; 3) some college; or 4) college completed, or some graduate or professional school. Income level was categorized as affluent, upper-middle, lower-middle, or poor based on total combined family income, family size, and poverty levels in each year when the interview was conducted. Some participants (9.5% and 13.8% in ARIC and JHS, respectively) did not provide their income information and were coded as a separate category (missing). A more detailed description of the assessment of income is presented in Text S1. Occupations were categorized according to the criteria of the 1980 U.S. census into six groups: managerial and professional specialty; sales, technical, and administrative support; service; farming, forestry and fishing or precision production; operators, fabricators, laborers or construction; and homemakers [54]. Because cohort controls had not been genotyped in the MEC (as we had oversampled particular phenotypes for genetic studies), the analyses of SES was limited to ARIC and JHS only.

### Genotyping

Participants were genotyped with at least one of three iteratively improved and partially overlapping panels of ancestry-informative SNP markers [23], [25], [41], [55], [56]. The ARIC study used the Phase 3 panel, the JHS study used Phase 2 and 3, and the MEC study used all three panels. Altogether 2,189 markers were genotyped in the present study, with a median of 1,243 markers per individual. We used previously published genotyping data to estimate the frequency of each SNP in West Africans and European Americans, the two parental populations of African Americans [20], [53], [55]. A series of filters, as described previously [25], [55]–[57], were applied to detect and remove SNPs with problematic genotyping. Genotyping details, estimates of allele frequencies, and SNP quality control checks are presented in Text S1.

### Estimating genetic ancestry

We estimated each subject's global percentage of African ancestry using the ANCESTRYMAP software [40]. ANCESTRYMAP uses a Hidden Markov Model (HMM) to combine the weak information about local ancestry that is provided by each SNP into a more confident estimate that incorporates information from many neighboring markers. Use of the HMM to estimate genetic ancestry is described in more detail in Text S1.

### Statistical analysis

Statistical analyses were performed using Stata 10.1 (Stata Corporation, College Station, Texas, US) and ANCESTRYMAP. Initial analyses of the correlation between African ancestry and either SES or the diabetes-related quantitative traits were performed using Spearman's correlation coefficient (ρ). Analyses of ancestry associations were conducted using pooled data from all three cohorts. Quantitative traits were available for analysis in ARIC and JHS only.

We used logistic regression models to estimate the OR for diabetes, comparing tertiles 2 and 3 to the lowest tertile of the distribution of African ancestry. To explore further potential nonlinear ancestry-diabetes associations, we used restricted cubic splines with equally-spaced knots at the 5th, 35th, 65th, and 95th percentiles of the ancestry distribution. For quantitative traits, we used linear regression models to determine the proportion of variation in trait levels explained by each variable (i.e., the coefficient of determination) and to assess the change in trait levels with ancestry tertiles. We constructed a series of multivariate models for our regression analyses. The base models were adjusted for age, sex and study only. Subsequent models were created by introducing BMI and SES as covariates, separately and collectively in sequence, because both SES and BMI correlate with ancestry [38], [41] and thus may potentially confound ancestry-diabetes association.

To quantify the extent to which groups of covariates appeared to explain the excess odds of diabetes with increasing African ancestry, we calculated the percentage reduction in the OR associated with adjustment (see Text S1 for more details). Similar calculation was performed to determine the relative contribution of covariates to the observed association between ancestry and quantitative traits.

We used ANCESTRYMAP [40] to search for genomic regions associated with an increased percentage of either European or African ancestry. The ANCESTRYMAP software provided two statistics: a *locus-genome statistic* and a *case-control statistic*. A *locus-genome statistic* was obtained in cases by calculating the likelihood of the genotyping data at the SNPs at the locus under a risk model and comparing it to the likelihood of the genotyping data at the SNPs at the locus assuming that the locus is unassociated with the phenotype [40]. We tested 6 pre-specified European ancestry risk models ranging from 0.7 to 1.3. To accumulate evidence of association in these models, we averaged the ratio of these two likelihoods emerging from each model at each point in the genome, taking the log_10_ of this likelihood ratio to produce a locus-specific LOD score. We considered a locus-specific LOD score for association at a particular locus of >5 as genome-wide significant and >4 as suggestive [58]. To obtain an assessment of the evidence for a risk locus anywhere in the genome, we averaged the likelihood ratio for association across all loci in the genome, and took the log10 to obtain a “genome-wide score” [58]. We interpreted a genome-wide score >2 as significant, and >1 as suggestive.

A *case-control statistic* was calculated by comparing locus-specific deviations in European ancestry in cases versus controls at each locus across the genome [40]. For loci identified by the case-control statistic, the level of genome-wide significance was defined as a Z score >4.06 or <−4.06, corresponding to an uncorrected nominal *P*<5×10^−5^, or a corrected nominal *P*<0.05 after conservatively correcting for 1,000 hypotheses tested (approximately equals the number of independent chromosomal segments assigned to either African or European ancestry).

## Supporting Information

Figure S1 **Histograms of African ancestry in the African American participants by study and diabetes status.** Percentages of African ancestry were estimated using subsets of 2,189 ancestry-informative SNPs. The grey bars represent the diabetic participants; the blue bars represent the non-diabetic participants.
(DOC)

Figure S2 **Scatterplot of hemoglobin A_1c_ and percentage of African ancestry in the ARIC and JHS studies.** The solid line in the figure is a lowess smoother.
(DOC)

Table S1 **Characteristics of ARIC and JHS study participants by diabetes status.**
(DOC)

Table S2 **Genetic African ancestry by socioeconomic status in African Americans in the ARIC and JHS studies (n = 5470).**
(DOC)

Table S3 **Association of diabetes and socioeconomic status in African Americans in the ARIC and JHS studies.**
(DOC)

Table S4 **Genetic African ancestry by diabetes-related quantitative traits in African Americans in the ARIC and JHS studies.**
(DOC)

Table S5 **Mean difference in the levels of diabetes-related quantitative traits by genetic African Ancestry.**
(DOC)

Table S6 **Summary of the Admixture scans on type 2 diabetes results by chromosome.**
(DOC)

Text S1 **Supplementary Methods: study populations, assessment of income, genotyping and quality filters, estimating genetic ancestry with Hidden Markov Models, and calculating contribution of covariates to the observed association.**
(DOC)
