# Correction: African Ancestry and Its Correlation to Type 2 Diabetes in African Americans: A Genetic Admixture Analysis in Three U.S. Population Cohorts

**DOI:** 10.1371/annotation/52e53d8e-a4bd-4419-951a-75ddcbaf6c5d

**Published:** 2012-09-20

**Authors:** Ching-Yu Cheng, David Reich, Christopher A. Haiman, Arti Tandon, Nick Patterson, Selvin Elizabeth, Ermeg L. Akylbekova, Frederick L. Brancati, Josef Coresh, Eric Boerwinkle, David Altshuler, Herman A. Taylor, Brian E. Henderson, James G. Wilson, W. H. Linda Kao

The sixth author's name was spelled incorrectly. The correct name is: Elizabeth Selvin.

