# Supplementary material for: African Ancestry and Its Correlation to Type 2 Diabetes in African Americans: A Genetic Admixture Analysis in Three U.S. Population Cohorts
Source: PLoS One. 2012 Mar 16;7(3):e32840. doi: 10.1371/journal.pone.0032840 (PMC3306373; doi:10.1371/journal.pone.0032840)
Supplement: Figure S1 — Histograms of African ancestry in the African American participants by study and diabetes status. Percentages of African ancestry were estimated using subsets of 2,189 ancestry-informative SNPs. The grey bars represent the diabetic participants; the blue bars represent the non-diabetic participants. (DOC) [file pone.0032840.s001.doc]

**Figure S1. Histograms of African ancestry in the African American participants by study and diabetes status.** Percentages of African ancestry were estimated using a subset of 2,189 ancestry-informative SNPs. The grey bars represent the diabetic participants; the blue bars represent the non-diabetic participants.

**ARIC**

**JHS**

**MEC**
