# Supplementary material for: African Ancestry and Its Correlation to Type 2 Diabetes in African Americans: A Genetic Admixture Analysis in Three U.S. Population Cohorts
Source: PLoS One. 2012 Mar 16;7(3):e32840. doi: 10.1371/journal.pone.0032840 (PMC3306373; doi:10.1371/journal.pone.0032840)
Supplement: Figure S2 — Scatterplot of hemoglobin A1c and percentage of African ancestry in the ARIC and JHS studies. The solid line in the figure is a lowess smoother. (DOC) [file pone.0032840.s002.doc]

**Figure S2. Scatterplot of hemoglobin A_1c_ and percentage of African ancestry in the ARIC and JHS studies.** The solid line in the figure is a lowess smoother.

N = 4880

ρ = 0.085

*P* < 0.001
