# Supplementary material for: African Ancestry and Its Correlation to Type 2 Diabetes in African Americans: A Genetic Admixture Analysis in Three U.S. Population Cohorts
Source: PLoS One. 2012 Mar 16;7(3):e32840. doi: 10.1371/journal.pone.0032840 (PMC3306373; doi:10.1371/journal.pone.0032840)
Supplement: Table S1 — Characteristics of ARIC and JHS study participants by diabetes status. (DOC) [file pone.0032840.s003.doc]

**Table S1.** Characteristics of ARIC and JHS study participants by diabetes status

|  | **ARIC** | | |  | **JHS** | | |
| --- | --- | --- | --- | --- | --- | --- | --- |
| **Characteristic** | **With Diabetes**  **(n = 631)** | **Without Diabetes**  **(n = 1654)** | ***P* Value** |  | **With Diabetes**  **(n = 829)** | **Without Diabetes**  **(n = 2356)** | ***P* Value** |
| Age | 55.6 ± 5.7 | 53.5 ± 5.9 | <0.001 |  | 60.9 ± 9.8 | 55.4 ± 11.6 | <0.001 |
| Gender |  |  |  |  |  |  |  |
| Men | 219 (34.7) | 699 (42.3) | 0.001 |  | 286 (34.5) | 924 (39.2) | 0.016 |
| Women | 412 (65.3) | 955 (57.7) |  |  | 543 (65.5) | 1432 (60.8) |  |
| BMI | 32.1 ± 6.2 | 28.5 ± 6.0 | <0.001 |  | 33.9 ± 6.9 | 31.3 ± 6.8 | <0.001 |
| Education level |  |  |  |  |  |  |  |
| ≥Bachelor degree | 98 (15.5) | 679 (41.1) |  |  | 224 (27.0) | 886 (37.6) |  |
| >High school but <bachelor degree | 92 (14.6) | 381 (23.0) | <0.001 |  | 202 (24.4) | 647 (27.5) | <0.001 |
| High school or GED | 120 (19.0) | 285 (17.2) |  |  | 166 (20.0) | 454 (19.3) |  |
| <High school | 321 (50.9) | 309 (18.7) |  |  | 237 (28.6) | 369 (15.7) |  |
| Family income |  |  |  |  |  |  |  |
| Affluent | 50 (7.9) | 213 (12.9) |  |  | 157 (18.9) | 707 (30.0) |  |
| Upper middle | 115 (18.2) | 412 (24.9) | <0.001 |  | 206 (24.8) | 617 (26.2) | <0.001 |
| Lower middle | 170 (26.9) | 398 (24.1) |  |  | 208 (25.1) | 468 (19.9) |  |
| Low | 240 (38.0) | 470 (28.4) |  |  | 132 (15.9) | 251 (10.7) |  |
| Occupation |  |  |  |  |  |  |  |
| Management | 108 (17.1) | 297 (18.0) |  |  | 259 (31.2) | 913 (38.8) |  |
| Sales | 59 (9.4) | 197 (11.9) |  |  | 117 (14.1) | 404 (17.2) |  |
| Service | 202 (32.0) | 440 (26.6) | <0.001 |  | 251 (30.3) | 563 (23.9) | <0.001 |
| Farming/production | 54 (8.6) | 205 (12.4) |  |  | 150 (18.1) | 320 (13.6) |  |
| Operators/construction | 111 (17.6) | 359 (21.7) |  |  | 47 (5.7) | 151 (6.4) |  |
| Homemaker | 97 (15.4) | 156 (9.4) |  |  | 5 (0.6) | 5 (0.2) |  |

ARIC, the Atherosclerosis Risk in Communities Study; JHS, the Jackson Heart Study; BMI, body mass index (calculated as weight in kilograms divided by height in meters squared); GED, high school-level General Educational Development credential.

Data are number (percentage, %), except age and BMI, which are expressed as mean ± standard deviation.
