# Supplementary material for: African Ancestry and Its Correlation to Type 2 Diabetes in African Americans: A Genetic Admixture Analysis in Three U.S. Population Cohorts
Source: PLoS One. 2012 Mar 16;7(3):e32840. doi: 10.1371/journal.pone.0032840 (PMC3306373; doi:10.1371/journal.pone.0032840)
Supplement: Table S2 — Genetic African ancestry by socioeconomic status in African Americans in the ARIC and JHS studies (n = 5470). (DOC) [file pone.0032840.s004.doc]

**Table S2.** Genetic African ancestry by socioeconomic status in African Americans in the ARIC and JHS studies (n = 5470).

| **Socioeconomic Status** | **No. (%)** | **African Ancestry, Median (IQR), %** | ***P* Value**^a^ |
| --- | --- | --- | --- |
| Education level |  |  |  |
| ≥Bachelor degree | 1517 (27.7) | 81.8 (74.0-87.5) |  |
| >High school but <Bachelor degree | 1226 (22.4) | 84.2 (78.6-88.7) | <0.001 |
| High school or GED | 1121 (20.5) | 84.6 (77.9-89.1) |  |
| <High school | 1606 (29.4) | 86.2 (80.5-90.1) |  |
| Family income^b^ |  |  |  |
| Affluent | 1127 (20.6) | 81.8 (74.1-87.7) |  |
| Upper middle | 1350 (24.7) | 84.0 (77.8-88.8) | <0.001 |
| Lower middle | 1244 (22.7) | 85.2 (78.7-89.4) |  |
| Low | 1093 (20.0) | 86.2 (79.9-90.0) |  |
| Occupation |  |  |  |
| Management | 1577 (17.7) | 82.5 (75.1-87.9) |  |
| Sales | 777 (11.2) | 83.2 (76.7-88.4) |  |
| Service | 1456 (28.1) | 85.6 (79.7-89.7) | <0.001 |
| Farming/production | 729 (11.3) | 84.9 (78.7-89.1) |  |
| Operators/construction | 668 (20.6) | 85.7 (79.5-90.0) |  |
| Homemaker | 263 (11.1) | 85.8 (77.5-89.4) |  |

ARIC, the Atherosclerosis Risk in Communities Study; JHS, the Jackson Heart Study; IQR, interquartile range; GED, high school-level General Educational Development credential.

^a^ *P* value was generated from the Kruskal-Wallis test.

^b^ Of the study participants, 12% did not provide their income information.
