# Supplementary material for: African Ancestry and Its Correlation to Type 2 Diabetes in African Americans: A Genetic Admixture Analysis in Three U.S. Population Cohorts
Source: PLoS One. 2012 Mar 16;7(3):e32840. doi: 10.1371/journal.pone.0032840 (PMC3306373; doi:10.1371/journal.pone.0032840)
Supplement: Table S3 — Association of diabetes and socioeconomic status in African Americans in the ARIC and JHS studies. (DOC) [file pone.0032840.s005.doc]

**Table S3.** Association of diabetes and socioeconomic status in African Americans in the ARIC and JHS studies.

| **Socioeconomic Status** | **With Diabetes**  **(n = 1460), No. (%)** | **Without Diabetes**  **(n = 4010), No. (%)** | **Odds Ratio (95% Confidence Interval)** | |
| --- | --- | --- | --- | --- |
|  |  |  | **Model 1**^a^ | **Model 2**^b^ |
| Education level |  |  |  |  |
| ≥Bachelor degree | 322 (22.1) | 1195 (29.8) | 1 [Reference] | 1 [Reference] |
| >High school but <bachelor degree | 294 (20.1) | 932 (23.2) | 1.24 (1.03-1.49)^c^ | 1.16 (0.93-1.43) |
| High school or GED | 286 (19.6) | 835 (20.8) | 1.14 (0.94-1.37) | 1.02 (0.80-1.29) |
| <High school | 558 (38.2) | 1048 (26.1) | 1.56 (1.31-1.86)^d^ | 1.32 (1.03-1.69)^c^ |
|  |  |  | *P* for trend <0.001 | *P* for trend = 0.045 |
| Family income |  |  |  |  |
| Affluent | 207 (14.2) | 920 (22.9) | 1 [Reference] | 1 [Reference] |
| Upper middle | 321 (22.0) | 1029 (25.7) | 1.36 (1.11-1.66)^c^ | 1.36 (1.10-1.67)^c^ |
| Lower middle | 378 (25.9) | 866 (21.6) | 1.64 (1.34-2.01)^d^ | 1.58 (1.26-1.97)^d^ |
| Low | 372 (25.5) | 721 (18.0) | 1.96 (1.59-2.41)^d^ | 1.80 (1.42-2.28)^d^ |
|  |  |  | *P* for trend <0.001 | *P* for trend <0.001 |
| Occupation |  |  |  |  |
| Management | 367 (25.1) | 1210 (30.2) | 1 [Reference] | 1 [Reference] |
| Sales | 176 (12.1) | 601 (15.0) | 0.97 (0.79-1.20) | 0.83 (0.66-1.04) |
| Service | 453 (31.0) | 1003 (25.1) | 1.25 (1.06-1.48)^c^ | 0.91 (0.73-1.14) |
| Farming/production | 204 (14.0) | 525 (13.1) | 1.20 (0.97-1.48) | 0.89 (0.69-1.15) |
| Operators/construction | 158 (10.8) | 510 (12.7) | 1.00 (0.79-1.26) | 0.74 (0.56-0.97)^c^ |
| Homemaker | 102 (7.0) | 161 (4.0) | 1.72 (1.28-2.32)^d^ | 1.21 (0.87-1.70) |

^a^ Model 1: odds ratio was adjusted for age, sex, and study.

^b^ Model 2: odds ratio was adjusted for age, sex, study, and the other two socioeconomic indicators.

^c^ *P* <0.05, as compared to the reference quartile.

^d^ *P* <0.001, as compared to the reference quartile.
