# Supplementary material for: African Ancestry and Its Correlation to Type 2 Diabetes in African Americans: A Genetic Admixture Analysis in Three U.S. Population Cohorts
Source: PLoS One. 2012 Mar 16;7(3):e32840. doi: 10.1371/journal.pone.0032840 (PMC3306373; doi:10.1371/journal.pone.0032840)
Supplement: Table S4 — Genetic African ancestry by diabetes-related quantitative traits in African Americans in the ARIC and JHS studies. (DOC) [file pone.0032840.s006.doc]

**Table S4.** Genetic African ancestry by diabetes-related quantitative traits in African Americans in the ARIC and JHS studies.

| **Trait** | **No. (%)**^a^ | **African Ancestry, Median (IQR), %** | ***P* Value**^b^ |
| --- | --- | --- | --- |
| Hemoglobin A_1c_, % |  |  |  |
| <5.7 | 2329 (47.7) | 83.8 (76.9-88.5) |  |
| 5.7-<6.5 | 1458 (29.9) | 84.2 (77.8-89.2) | <0.001 |
| ≥6.5 | 1093 (22.4) | 85.6 (79.5-89.6) |  |
| Fasting glucose, mg/dL |  |  |  |
| <100 | 3037 (60.3) | 83.9 (77.6-88.7) |  |
| 100-<126 | 1348 (26.8) | 84.6 (77.3-89.1) | 0.011 |
| ≥126 | 652 (12.9) | 85.0 (79.3-89.4) |  |
| Fasting insulin, mU/L |  |  |  |
| Tertile 1 (<12) | 1920 (38.1) | 84.2 (77.0-89.2) |  |
| Tertile 2 (12-<19) | 1564 (31.1) | 83.9 (77.7-88.8) | 0.308 |
| Tertile 3 (≥19) | 1553 (30.8) | 84.7 (78.5-89.0) |  |
| HOMA-IR |  |  |  |
| Tertile 1 (<2.52) | 1684 (33.4) | 84.0 (77.0-89.2) |  |
| Tertile 2 (2.52-<4.55) | 1678 (33.3) | 84.1 (77.6-88.9) | 0.224 |
| Tertile 3 (≥4.55) | 1675 (33.3) | 84.7 (78.5-89.0) |  |

ARIC, the Atherosclerosis Risk in Communities Study; JHS, the Jackson Heart Study; HOMA-IR, homeostasis model assessment–estimated insulin resistance (calculated as fasting plasma glucose [mmol/l] times fasting serum insulin [mU/L] divided by 22.5).

^a^ A total of 4,880 participants had data on hemoglobin A_1c_, and 5,037 had complete data on fasting glucose, insulin and HOMA-IR.

^b^ *P* value was generated from the Kruskal-Wallis test.
