# Supplementary material for: African Ancestry and Its Correlation to Type 2 Diabetes in African Americans: A Genetic Admixture Analysis in Three U.S. Population Cohorts
Source: PLoS One. 2012 Mar 16;7(3):e32840. doi: 10.1371/journal.pone.0032840 (PMC3306373; doi:10.1371/journal.pone.0032840)
Supplement: Table S5 — Mean difference in the levels of diabetes-related quantitative traits by genetic African Ancestry. (DOC) [file pone.0032840.s007.doc]

**Table S5.** Mean difference in the levels of diabetes-related quantitative traits by genetic African ancestry

| **Trait and Model** | **African Ancestry^a^** | | |  | **Effect Explained, %^b^** | |
| --- | --- | --- | --- | --- | --- | --- |
|  | **Tertile 1** | **Tertile 2** | **Tertile 3** | ***P* Value for Trend** | **Tertile 2** | **Tertile 3** |
| Hemoglobin A_1c_, % (n=4880) |  |  |  |  |  |  |
| Model 1, base^c^ | 0 [Reference] | 0.18 (0.07-0.28)^g^ | 0.24 (0.13-0.35)^h^ | <0.001 | [Reference] | [Reference] |
| Model 2, BMI^d^ | 0 [Reference] | 0.15 (0.05-0.26)^g^ | 0.21 (0.11-0.31)^h^ | <0.001 | 16.7 | 12.5 |
| Model 3, SES^e^ | 0 [Reference] | 0.13 (0.03-0.24)^g^ | 0.18 (0.08-0.29)^g^ | 0.001 | 27.8 | 25.0 |
| Model 4, BMI + SES^f^ | 0 [Reference] | 0.12 (0.01-0.22)^g^ | 0.16 (0.06-0.27)^g^ | 0.002 | 33.3 | 33.3 |
| Glucose, mg/dL (n=5037) |  |  |  |  |  |  |
| Model 1, base^c^ | 0 [Reference] | 2.56 (–0.25-5.38) | 2.84 (0.01-5.66)^g^ | 0.048 | [Reference] | [Reference] |
| Model 2, BMI^d^ | 0 [Reference] | 2.07 (–0.72-4.86) | 2.14 (–0.66-4.94) | 0.133 | 19.1 | 24.6 |
| Model 3, SES^e^ | 0 [Reference] | 1.34 (–1.50-4.17) | 1.31 (–1.55-4.18) | 0.369 | 47.7 | 53.9 |
| Model 4, BMI + SES^f^ | 0 [Reference] | 1.00 (–1.81-3.80) | 0.82 (–2.02-3.65) | 0.574 | 60.9 | 71.1 |
| Insulin, mU/L (n=5037) |  |  |  |  |  |  |
| Model 1, base^c^ | 0 [Reference] | 1.05 (–0.73-2.84) | 1.20 (–0.59-2.99) | 0.188 | [Reference] | [Reference] |
| Model 2, BMI^d^ | 0 [Reference] | 0.63 (–1.12-2.38) | 0.60 (–1.16-2.36) | 0.501 | 40.0 | 50.0 |
| Model 3, SES^e^ | 0 [Reference] | 0.87 (–0.93-2.68) | 0.99 (–0.84-2.81) | 0.289 | 17.1 | 17.5 |
| Model 4, BMI + SES^f^ | 0 [Reference] | 0.57 (–1.20-2.34) | 0.54 (–1.25-2.33) | 0.555 | 45.7 | 55.0 |
| HOMA-IR (n=5037) |  |  |  |  |  |  |
| Model 1, base^c^ | 0 [Reference] | 0.47 (–0.26-1.20) | 0.59 (–0.14-1.32) | 0.110 | [Reference] | [Reference] |
| Model 2, BMI^d^ | 0 [Reference] | 0.33 (–0.39-1.05) | 0.40 (–0.32-1.12) | 0.279 | 29.4 | 32.2 |
| Model 3, SES^e^ | 0 [Reference] | 0.35 (–0.39-1.08) | 0.44 (–0.30-1.18) | 0.246 | 25.9 | 25.4 |
| Model 4, BMI + SES^f^ | 0 [Reference] | 0.25 (–0.48-0.98) | 0.30 (–0.44-1.03) | 0.431 | 47.0 | 49.2 |

BMI, body mass index (calculated as weight in kilograms divided by height in meters squared); SES, socioeconomic status (including education, income and occupation); CI, confidence interval.

^a^ Tertiles 1, 2 and 3 of African ancestry are <80.6%, 80.6%-87.5% and >87.5%, respectively, for hemoglobin A_1c_, and <80.4%, 80.4% -87.4% and >87.4%, respectively, for glucose, insulin, and HOMA-IR.

^b^Effects explained is defined as (β_1_ – β_2_)/β_1_ where β_1_ is the regression coefficient of traits in Model 1; β_2_ is the regression coefficient after additional adjustment for covariates in each model.

^c^ Model 1: Mean difference (95% confidence interval) is adjusted for age, sex, and study.

^d^ Model 2: Mean difference (95% confidence interval) is adjusted for covariates in Model 1 and BMI.

^e^ Model 3: Mean difference (95% confidence interval) is adjusted for covariates in Model 1 and SES.

^f^ Model 4: Mean difference (95% confidence interval) is adjusted for covariates in Model 1, BMI, and SES.

^g^ *P*<0.05, as compared to the reference tertile.

^h^ *P*<0.001, as compared to the reference tertile.
