# Supplementary material for: African Ancestry and Its Correlation to Type 2 Diabetes in African Americans: A Genetic Admixture Analysis in Three U.S. Population Cohorts
Source: PLoS One. 2012 Mar 16;7(3):e32840. doi: 10.1371/journal.pone.0032840 (PMC3306373; doi:10.1371/journal.pone.0032840)
Supplement: Table S6 — Summary of the Admixture scans on type 2 diabetes results by chromosome. (DOC) [file pone.0032840.s008.doc]

**Table S6.** Summary of the admixture scans on type 2 diabetes results by chromosome

| **Chromosome** | **Highest Locus-Specific LOD score** | **Highest Case-Control Z score** | **Lowest Case-Control Z score** |
| --- | --- | --- | --- |
| 1 | 3.5 | 3.1 | -2.4 |
| 2 | 0.7 | 2.7 | -1.5 |
| 3 | 0.2 | 2.9 | -2.0 |
| 4 | 0.7 | 2.0 | -1.6 |
| 5 | 1.2 | 2.9 | -2.6 |
| 6 | 0.0 | 0.9 | -1.4 |
| 7 | 0.4 | 0.6 | -2.7 |
| 8 | 2.5 | 2.5 | -0.7 |
| 9 | 0.4 | 1.2 | -1.1 |
| 10 | -0.2 | 2.6 | -1.5 |
| 11 | 0.2 | 1.9 | -2.3 |
| 12 | 4.0 | 0.1 | -4.2 |
| 13 | 1.1 | 4.5 | -0.6 |
| 14 | -0.3 | 1.7 | -1.4 |
| 15 | 1.0 | 1.8 | -2.0 |
| 16 | 1.0 | 0.2 | -3.1 |
| 17 | 0.6 | 2.5 | -1.0 |
| 18 | 1.4 | 0.9 | -2.3 |
| 19 | -0.7 | 1.6 | -1.8 |
| 20 | 0.7 | 2.3 | -0.8 |
| 21 | -0.2 | 0.7 | -0.9 |
| 22 | 0.5 | 1.5 | 0.1 |
| X | 1.5 | 2.1 | -2.8 |
