# Supplementary material for: African Ancestry and Its Correlation to Type 2 Diabetes in African Americans: A Genetic Admixture Analysis in Three U.S. Population Cohorts
Source: PLoS One. 2012 Mar 16;7(3):e32840. doi: 10.1371/journal.pone.0032840 (PMC3306373; doi:10.1371/journal.pone.0032840)
Supplement: Text S1 — Supplementary Methods: study populations, assessment of income, genotyping and quality filters, estimating genetic ancestry with Hidden Markov Models, and calculating contribution of covariates to the observed association. (DOC) [file pone.0032840.s009.doc]

**Supporting Information**

Cheng CY, Reich D, Haiman CA, et al. African Ancestry and its Correlation to Type 2 Diabetes in African Americans: A Genetic Admixture Analysis in Three U.S. Population Cohorts.

**Text S1. Supplementary Methods**

**Study populations**

The ARIC study is a prospective, epidemiologic study that examines clinical and subclinical atherosclerotic disease in a cohort of 15,792 persons, including 4,266 self-reported African Americans, aged 45 to 64 years at their baseline examination from 1987 to 1989. Participants were selected by probability sampling from four communities: Forsyth County, NC (12% African American); Jackson, MS (100% African American); the northwest suburbs of Minneapolis, MN (<1% African American); and Washington County, MD (<1% African American). The sampling procedure and methods used in ARIC have been described in detail elsewhere [1]. Of the African Americans, 1,626 Jackson-based participants were later enrolled in the JHS study. For the present analysis, a total of 3,436 African Americans had known status of type 2 diabetes and measurements of BMI, and were successfully genotyped with ancestry-informative markers. Of these, there were 2,310 “ARIC unique” participants who were present only in the ARIC study, and 1,126 were “JHS-ARIC overlap” participants, representing persons who had participated in both the JHS and ARIC study. We further excluded 22 who had missing education or occupation information from the “ARIC unique” group and 3 who had relatives being included in the “JHS unique” group, leaving 2,285 that are unique to the ARIC study for our analysis.

The JHS study is a long-term, community-based observational study of cardiovascular disease and its risk factors in 5,301 self-identified African Americans recruited between 2000 and 2004 from three counties surrounding Jackson, Mississippi [2,3]. Unrelated persons aged 35–84 were enrolled from three sources: previous ARIC participants (31% of the total), random selectees from a commercial listing (17%), and members of an age and sex-constrained volunteer sample (30%). The remaining participants, at least 21 years old, were members of the nested JHS Family Study [4]. A total of 4,389 participants with known status of type 2 diabetes and BMI measurements were successfully genotyped. Of these, 3,263 were “JHS unique” participants that were present only in the JHS study, and 1,126 were “JHS-ARIC overlap” participants as described above. A subset of 3,228 was left after we preferentially kept diabetic participants and randomly dropped related individuals until there was only one individual included per family. For those in the “JHS-ARIC overlap” group, we used the baseline measurements at the time of their entry into the JHS study, an average of 14 years after the ARIC study. We further excluded 41 participants for missing either education or occupation information, and another 7 individuals for having excess heterozygous genotypes, which can be an indication of sample contamination or be representative of individuals who have parents with divergent ancestries (e.g., one parent who is entirely of European ancestry and another who is entirely of African ancestry), leaving 3,185 participants from the JHS study for this analysis.

The MEC study is a prospective cohort that includes over 215,251 individuals recruited between 1993 and 1996 in Hawaii and Los Angeles, California, of whom 16.3% are African Americans [5]. Potential cohort members were identified primarily through Department of Motor Vehicles drivers’ license files and, additionally for African Americans, Health Care Financing Administration data files. Participants were between the ages of 45 and 75 years at the time of recruitment. The MEC participants in this analysis were not a random cross-section of the population; instead, they included an oversampling of individuals with prostate cancer, hypertension and diabetes, whom we had admixture scanned in other studies focusing on the phenotypes [6,7]. There were 1,559 African Americans in MEC with information on diabetes status and BMI and were successfully genotyped. Five subjects with an apparent excess of heterozygous genotypes and three with an estimated African ancestry <15% (see “Estimating Genetic Ancestry” in the Methods) were excluded, leaving 1,551 from the MEC study.

**Assessment of income**

The ARIC and JHS study used a similar protocol to collect information on participants’ income. In ARIC, participants were asked to select their combined family income from a list of 8 categories: less than $5,000, $5,000 to 7,999, $8,000 to 11,999, $12,000 to $15,999, $16,000 to $24,999, $25,000 to $34,999, $35,000 to $49,999, and $50,000 and more. In JHS, participants were asked to select their combined family income from a list of 11 categories, which can be collapsed into the 8 categories as used in ARIC. Information in family size (number of persons in household) was also collected during the interview.

Income level was assigned and classified into 4 groups based on the combined family income, family size, and poverty thresholds in each year (<http://www.census.gov/hhes/www/poverty/>) when the interview was conducted. For example, in the ARIC study for a two and three person family in 1990, the income level was assigned as poor (family income ≤$7,999), lower middle ($8,000 to $ 15,999), upper middle ($16,000 to $ 34,999), or affluent (≥ $35,000). In JHS, for a same size family in 2000, the income level was assigned as poor (≤$11,999), lower middle ($12,000 to $ 24,999), upper middle ($25,000 to $ 49,999), or affluent (≥ $50,000).

**Genotyping and quality filters**

Study participants were genotyped with at least one of three panels of ancestry-informative SNP markers, including the Phase 1 [8,9], 2 [10], and 3 [11,12] panels. The genotyping for the ARIC study was carried out using genomic DNA on the Phase 3 marker panel at the Center for Inherited Disease Research, Johns Hopkins University. The genotyping for the JHS study was carried out using genomic DNA on either the Phase 2 or Phase 3 marker panels, at the Broad Institute of Harvard and MIT. The genotyping for the MEC study was done using genomic DNA on one of the three panels at the Broad Institute of Harvard and MIT and the University of Southern California.

Previously published genotyping data were used to estimate the frequency of each SNP in the two parental populations of African Americans [6–8]. We only used SNPs for which we were able to obtain data from both West African Yoruba (YRI) and European American (CEU) populations from the International HapMap Project. For SNPs in the Phase 1 panel, we also included additional genotyping data from African and European samples, as described previously [8].

We applied a series of filters to detect and remove SNPs with problematic genotyping [8–10]. First, we applied a “mapcheck” filter that tested whether the ancestry estimate obtained based on the information from that SNP alone was consistent with the estimate obtained from neighboring markers, and SNPs with discrepancies were removed from analysis. Second, we applied a “freqcheck” filter that tested whether the observed frequency of a SNP in African Americans was statistically consistent with being a mixture of the frequencies observed in the West African and European American samples that we used to represent the ancestral populations. Finally we applied an “ldcheck” filter that, for each subject, iteratively removed SNPs that were less informative about ancestry until none were within 200 kilobases of each other or are in detectable linkage disequilibrium with each other in the ancestral West African or European American populations [6,9].

We also removed participants with low quality genotype data (genotyping rate for each sample was < 90%). The data for the individuals we analyzed in this study were reported previously [6,7,10–13], and hence we do not report further details on elimination of problematic samples here.

**Estimating genetic ancestry with Hidden Markov Models**

The Hidden Markov Model in the ANCESTRYMAP software is nested within a Markov Chain Monte Carlo [14] to account for uncertainty in the unknown parameters: SNP allele frequencies in the West African and European American ancestral populations, the number of generations since mixture, and the average proportion of ancestry inherited from ancestral populations. All Markov Chain Monte Carlo runs used 100 burn-in and 200 follow-on iterations, as recommended [14].

**Calculating contribution of covariates to the observed association**

To quantify the extent to which groups of covariates appeared to explain the excess odds of diabetes with increasing African ancestry, we calculated the percentage reduction in the odds ratio (OR) associated with adjustment, according to the formula: percentage reduction = (θ_1_ – θ_2_)/(θ_1_ − 1), where θ _1_ is the OR of diabetes due to increase in African ancestry in the base model, adjusted for age, sex and study; θ_2_ is the OR after additional adjustment for other covariates; and θ_1_ −1 is the excess odds of diabetes due to increase in African ancestry. Similarly, to determine the relative contribution of covariates to the observed association between ancestry and quantitative traits, we used the formula: (β_1_ – β_2_)/β_1_, where β_1_ is the linear regression coefficient in the base model and β_2_ is the regression coefficient after further adjustment for covariates.

**References**

1. The ARIC Investigators (1989) The Atherosclerosis Risk in Communities (ARIC) Study: design and objectives. The ARIC investigators. Am J Epidemiol 129: 687-702.

2. Taylor HA, Jr. (2003) Establishing a foundation for cardiovascular disease research in an African-American community--the Jackson Heart Study. Ethn Dis 13: 411-413.

3. Taylor HA, Jr., Wilson JG, Jones DW, Sarpong DF, Srinivasan A et al. (2005) Toward resolution of cardiovascular health disparities in African Americans: design and methods of the Jackson Heart Study. Ethn Dis 15: S6-17.

4. Wilson JG, Rotimi CN, Ekunwe L, Royal CD, Crump ME et al. (2005) Study design for genetic analysis in the Jackson Heart Study. Ethn Dis 15: S6-37.

5. Kolonel LN, Henderson BE, Hankin JH, Nomura AM, Wilkens LR et al. (2000) A multiethnic cohort in Hawaii and Los Angeles: baseline characteristics. Am J Epidemiol 151: 346-357.

6. Freedman ML, Haiman CA, Patterson N, McDonald GJ, Tandon A et al. (2006) Admixture mapping identifies 8q24 as a prostate cancer risk locus in African-American men. Proc Natl Acad Sci U S A 103: 14068-14073.

7. Deo RC, Patterson N, Tandon A, McDonald GJ, Haiman CA et al. (2007) A High-Density Admixture Scan in 1,670 African Americans with Hypertension. PLoS Genet 3: e196.

8. Smith MW, Patterson N, Lautenberger JA, Truelove AL, McDonald GJ et al. (2004) A high-density admixture map for disease gene discovery in African Americans. Am J Hum Genet 74: 1001-1013.

9. Reich D, Patterson N, De Jager PL, McDonald GJ, Waliszewska A et al. (2005) A whole-genome admixture scan finds a candidate locus for multiple sclerosis susceptibility. Nat Genet 37: 1113-1118.

10. Reich D, Patterson N, Ramesh V, De Jager PL, McDonald GJ et al. (2007) Admixture mapping of an allele affecting interleukin 6 soluble receptor and interleukin 6 levels. Am J Hum Genet 80: 716-726.

11. Nalls MA, Wilson JG, Patterson NJ, Tandon A, Zmuda JM et al. (2008) Admixture mapping of white cell count: genetic locus responsible for lower white blood cell count in the Health ABC and Jackson Heart studies. Am J Hum Genet 82: 81-87.

12. Cheng CY, Reich D, Coresh J, Boerwinkle E, Patterson N et al. (2010) Admixture mapping of obesity-related traits in African Americans: the Atherosclerosis Risk in Communities (ARIC) Study. Obesity (Silver Spring) 18: 563-572.

13. Kao WH, Klag MJ, Meoni LA, Reich D, Berthier-Schaad Y et al. (2008) MYH9 is associated with nondiabetic end-stage renal disease in African Americans. Nat Genet 40: 1185-1192.

14. Patterson N, Hattangadi N, Lane B, Lohmueller KE, Hafler DA et al. (2004) Methods for high-density admixture mapping of disease genes. Am J Hum Genet 74: 979-1000.
